# Factors affecting fat myringoplasty in elderly patients with chronic otitis media: A case control study

**DOI:** 10.1186/s40001-024-02085-y

**Published:** 2024-10-03

**Authors:** Kyeong Suk Park, Ji Su Kim, Chung Man Sung, Hyong Ho Cho, Hong Chan Kim

**Affiliations:** 1 Department of Otolaryngology-Head and Neck Surgery, Chonnam National University Medical School and Chonnam National University Hospital, 160 Baekseo-Ro, Dong-Gu , Gwangju, 61469 Republic of Korea; 2 Department of Biomedical Science and Biomedical Graduate Program (BMSGP), Chonnam National University Medical School, Gwangju, Republic of Korea

**Keywords:** Fat myringoplasty, Chronic otitis media, Hearing aid, Presbycusis

## Abstract

**Background:**

We compared and analyzed the surgical results of fat myringoplasty between elderly and young adult patients with chronic otitis media. We also investigated whether underlying diseases and other factors impact the surgical outcome.

**Methods:**

We retrospectively reviewed the data of 141 patients who underwent fat myringoplasty for chronic otitis media for five years. They were compared by age, sex, underlying disease, perforation size, pre- and postoperative pure tone audiometry, postoperative otorrhea, postoperative re-perforation, and cause of re-perforation.

**Result:**

Postoperative re-perforation was more common in the elderly group, albeit with no significant difference (p = 0.072). The factors affecting re-perforation were insufficient fat graft (44.4%), postoperative infection (33.3%), and nasal blowing (22.2%). Our findings revealed no significant association between preoperative perforation size and re-perforation (p = 0.391). Additionally, we found no significant relationship between hypertension and re-perforation (p > 0.99), nor between age group and postoperative infection (p = 0.488). Diabetes was also not significant (p = 0.640). Following surgery, both groups exhibited a significant improvement in hearing.

**Conclusion:**

Although age and underlying conditions play significant roles in the healing process, our results suggest that external factors such as infection, nasal blowing, cough, and insufficient grafted fat tissue have a similarly significant impact on surgical outcomes in elderly patients with COM as they do in adults. In conclusion, the decision to perform surgery in elderly patients with COM should be based on a comprehensive assessment of the patient’s overall health status, hearing, use of hearing aids, and the indications for surgery.

## Introduction

In Korea, the population aged ≥ 65 years has been rapidly increasing, comprising over 14% of the total population in 2018, with projections indicating a rise to more than 20% by 2025 [1]. Therefore, social interest in geriatric diseases is increasing, and the health problems of older people are being emphasized in public health care. Additionally, the elderly actively contribute to the treatment of disease to improve their quality of life and ability to participate in social activities.

Chronic otitis media (COM), a common disease of the ear in the elderly, is defined as long-standing inflammation of the middle ear leading to persistent perforation of the tympanic membrane [2]. Although the incidence of COM in Korea has been decreasing due to improvements in the medical environment and the development of antibiotics, many older people still suffer from hearing loss or ear discharge. Although increasing age is the major cause of hearing loss in older people, it can also be related to the duration of COM. Indeed, continuous exposure of the tympanic cavity to the external environment through tympanic membrane perforation can cause inflammation or damage to the inner ear, eventually leading to permanent hearing loss [3]. Besides, many older people with COM have to wear hearing aids due to hearing loss. Hearing aids may cause persistent ear discharge and bacterial or fungal infection due to occlusion of the ear canal by hearing aids. Therefore, patients with COM may require surgical management.

Bocca E [4] suggested that the patient’s age was an important factor in the failure of surgery for COM, while Tos M [5] showed that the surgical outcomes for COM were the worst in patients > 60 years old. Therefore, before the 1980s, surgery for COM was not recommended in the elderly owing to the high graft failure rate and the risk of complications. However, more recently, surgery may be advised for elderly patients with COM if the physical status of the patient is equal to or better than that considered normal for their chronological age [6]. Indeed, surgery for COM in elderly patients seems to be acceptable worldwide due to advances in surgical and anesthesia techniques, as well as the increase in the number of healthy older people, an increase in life expectancy, and an increase in patients’ desire for effective treatment.

Despite this, the effectiveness and safety of surgery for COM in older people remains controversial, with relatively few published papers on the topic, including only one report on the use of fat myringoplasty as a minimally invasive procedure for COM repair [7].

Therefore, in this study, we compared and analyzed the surgical results of fat myringoplasty between elderly and young adult patients with COM. We also investigated whether underlying diseases and other factors impact the surgical outcome.

## Materials and methods

### Participants

We retrospectively reviewed the data of 141 patients who underwent fat myringoplasty for COM in our tertiary hospital from January 2015 to December 2019. The patients were followed up for at least 6 months after the operation. The inclusion criteria were as follows: time elapsed from previous surgery ≥ 6 months; perforation size < 50% of the tympanic membrane by otoscopy; pneumatic mastoid on the temporal bone CT; no involvement of the annulus or no exposure of the malleus handle; and an air–bone gap > 25 dB. Additionally, patients who had acute ear inflammation, middle ear discharge in the last 3 months, evidence of cholesteatoma, major Eustachian tube dysfunction, and those who required ossicular reconstruction were excluded. The otoendoscopic images, pure tone audiometry (PTA) data, operation records, and medical history records of the included patients were investigated in detail.

### Methods

Surgical success was defined based on the condition of the tympanic membrane, with no consideration given to hearing improvement. A successful outcome was characterized by the presence of an intact, epithelialized neodrum without any perforation or retraction. All patients were followed up for at least 6 months, and surgical success was determined by endoscopic imaging.

Conventionally, “elderly” has been defined as a chronological age of 65 years old or older, while those from 65 through 74 years old are referred to as “early elderly” and those over 75 years old as “late elderly” [8]. Therefore, we classified the patients into two groups according to age: the adult group, aged 20–64 years, and the elderly group, aged ≥ 65 years. We compared the two groups by age, sex, underlying disease, perforation size, pre- and postoperative PTA, postoperative otorrhea, postoperative re-perforation, and cause of re-perforation. Furthermore, to identify hearing changes with age, we investigated the PTA in patients aged 20–29, 30–39, 40–49, 50–59, 60–69, 70–79, and 80 to 90 years. The average PTA was calculated as follows: average PTA = (0.5 kHz + 1 kHz + 2 kHz + 3 kHz) / 4, which is the international accepted method to calculate hearing [9].

### Surgical procedure (Fig. 1)

**Fig. 1:**
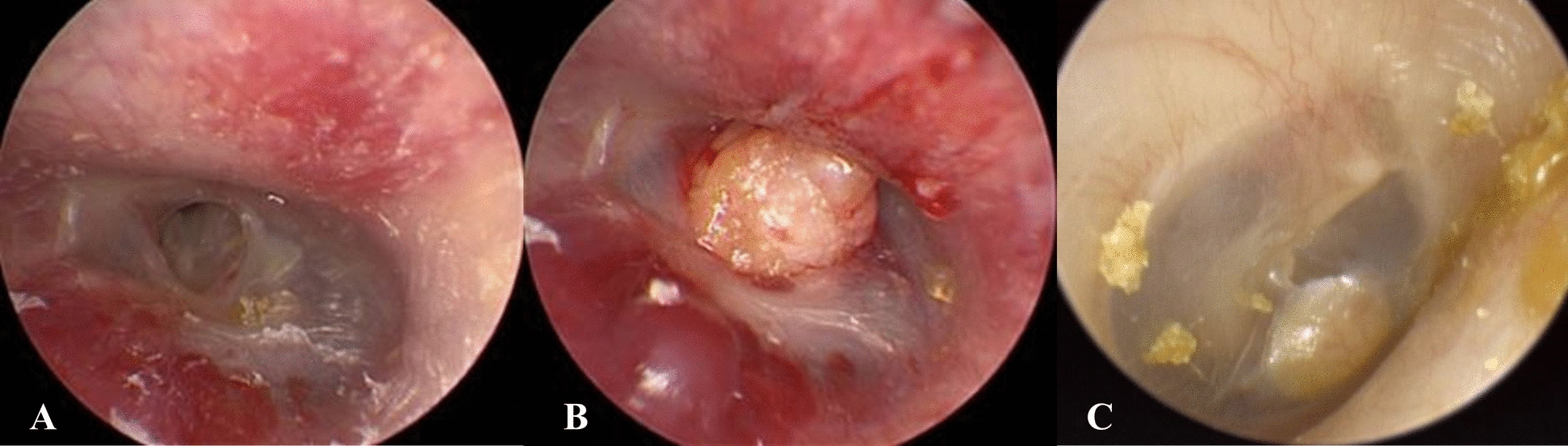
Pre-operative and post-operative photos of fat myringoplasty. A The margins of the tympanic membrane perforation were trimmed circumferentially under endoscope. B The harvested fat tissue was inserted through the perforation as an hourglass. C The tympanic membrane photo 8 months after surgery

The fat tissue for myringoplasty was obtained from the ear lobe on the same side as the operated ear. Lidocaine with 1:100,000 (w/w) epinephrine was infiltrated into the four quadrants of the osteocartilaginous junction of the external ear canal. The perforation margin was trimmed using a Rosen needle and removed using microforceps. Gelfoam soaked in ciprofloxacin and dexamethasone was inserted through the perforation into the middle ear, before placing the fat tissue on the Gelfoam (covering the perforation) and shaping to resemble an hourglass. The external ear canal was packed with Gelfoam soaked in antibiotic solution. Antibiotics were administered for one week post-operatively, and a follow-up appointment was conducted one week later. If an infection was suspected post-operatively, ear culture and ear dressing were performed.

### Statistical analysis

The obtained data were analyzed by chi-square, Fisher’s exact, and independent t-tests (IBM SPSS Statistics ver. 20; IBM Corp., Armonk, NY, USA). p-values < 0.05 were considered to indicate statistical significance.

## Results

### Demographics (Table 1)

**Table 1:** Baseline characteristics of the study population

Group/Parameter		Elderly (n = 51)	Adult (n = 90)	Total (n = 141)	P-value
Male/female		20/31	34/56	54/87	0.866^†^
Underlying disease	Hypertension (HTN)	33	14	47 (52%)	< 0.001^†^*
	Diabetes mellitus (DM)	9	14	23 (25%)	0.747^†^
	Arrhythmia, old CVA, old MI	4	5	9 (3%)	0.723^††^
	Asthma	2	4	6 (18%)	1.0^††^
Perforation size	Pinpoint (< 10%)	9	21	30 (21%)	0.019^†^*
	Small (10%–25%)	25	57	82 (58%)	
	Moderate (25%–50%)	17	12	29 (21%)	
Preoperative pure-tone audiometry (dB, mean)	Bone conduction threshold	47.5	19.1	29.4	< 0.001^†††^*
	Air conduction threshold	64.8	33.2	44.6	< 0.001^†††^*
	Air–bone gap	17.3	14.1	15.2	0.008^†††^*

^†^Chi-square test, ††Fisher’s exact test, †††independent t-test, *statistically significant

(n = number of cases)

CVA: cerebrovascular accident

MI: myocardial infarction

The study population consisted of 54 male and 87 female patients with a mean age of 59.0 years. We included 51 elderly patients aged 65 years or older and 90 control adult patients aged 20–64 years. The proportion of patients with hypertension was significantly higher in the elderly group compared to the control group. Nine patients had diabetes in the elderly group compared to 14 patients in the control group, with no significant difference. The proportion of patients with other diseases did not differ significantly between the two groups.

Perforation size was classified into pinpoint, small, and moderate according to the size ratio. The size of the perforation for fat myringoplasty was significantly different between the two groups. When comparing the pre-operative PTA, the bone conduction (BC), air conduction (AC), and air–bone gap (ABG) were all significantly higher in the elderly group.

### Surgical outcome (Table 2)

**Table 2:** Reperforation rate by previous perforation size, age, and underlying disease

Group/Parameter			Postoperative status		P-value
			Reperforation	No perforation	
Perforation size (%)		Pinpoint and small (< 25)	6	106	0.391^††^
		Moderate (25–50)	3	26	
Age (years)		Elderly (≥ 65)	6	45	0.072^††^
		Controls (adult, 20–64)	3	87	
Underlying disease	Hypertension	+	3	44	> 0.99^††^
		−	6	88	
	Diabetes mellitus	+	2	21	0.640^††^
		−	7	111	

^††^Fisher’s exact test, *statistically significant

(n = number of cases)

The postoperative occurrence of re-perforation, the most important factor in fat myringoplasty surgery, was compared according to differences in perforation size, age group, and the presence or absence of hypertension and diabetes mellitus among underlying diseases. In the pre-operative pinpoint and small size groups, six postoperative reperforations occurred, and three reperforations occurred in the moderate size group. The preoperative perforation size and postoperative reperforation did not differ significantly between the two groups. Next, six cases of reperforation occurred in the elderly group, and three cases occurred in the control group. Although reperforation was more common in the elderly group, the difference was not significant. Additionally, the relationship between hypertension and reperforation was not statistically significant. Diabetes was also not significant.

### Postoperative infection

Nine patients (6.4%) suffered postoperative infection after fat myringoplasty, including two in the elderly group and seven in the adult group. The relationship between age group and postoperative infection was not statistically significant (Table 3). Additionally, postoperative infection was not associated with sex, perforation size, and underlying disease (e.g., HTN, DM).

**Table 3:** Postoperative discharge rate by age

Group/Parameter		Age (years)		P-value
		Elderly (≥ 65)	Adult (20–64)	
Postoperative status	Discharge	2	7	0.488^††^
	No discharge	49	83	

^††^Fisher’s exact test, *statistically significant

(n = number of cases)

We performed culture tests on seven of the nine patients with postoperative infection. The results revealed the presence of methicillin-resistant *Staphylococcus aureus* (MRSA) in most cases (71.4%, 5/7). Additionally, re-perforation occurred in 60% (3/5) of the patients who experienced postoperative MRSA infection (Table 4).

**Table 4:** Discharge culture test results in patients with postoperative ear discharge

Cases	Sex	Age (y)	HTN	DM	Perforation size	Reperforation	Culture results
1	Female	38	−	−	Small	−	Methicillin-resistant *Staphylococcus aureus*
2	Male	39	−	−	Pin-point	−	Methicillin-resistant *Staphylococcus aureus*
3	Female	46	−	−	Pin-point	+	Methicillin-resistant *Staphylococcus aureus*
4	Female	50	−	−	Small	−	No culture test
5	Female	60	+	−	Moderate	+	Methicillin-resistant *Staphylococcus aureus*
6	Female	61	−	−	Small	−	*Staphylococcus hominis*
7	Male	62	−	−	Pin-point	−	No culture test
8	Male	68	+	+	Small	+	Methicillin-resistant *Staphylococcus aureus*
9	Female	79	+	−	small	−	No growth

### Cause of surgical failure (Table 5)

**Table 5:** Cases of surgical failure

Cases	Sex	Age (y)	HTN	DM	Perforation size	Postoperative infection	Cause
1	Male	35	−	−	Small	−	Fat extrusion due to nasal blowing or coughing
2	Female	46	−	−	Pin-point	+	MRSA infection
3	Female	60	+	−	Moderate	+	MRSA infection
4	Male	68	+	+	Small	+	MRSA infection
5	Female	68	−	−	Pin-point	−	Anterior dehiscence due to lack of fat graft
6	Male	69	+	+	Small	−	Anterior dehiscence due to lack of fat graft
7	Female	71	−	−	Moderate	−	Fat extrusion due to nasal blowing or coughing
8	Male	78	−	−	Small	−	Posterior dehiscence due to lack of fat graft
9	Female	78	−	−	Moderate	−	Anterior dehiscence due to lack of fat graft

In our study, re-perforation occurred in 9 of 141 (6.4%) patients. We analyzed various factors that may have affected the surgical results. As a result, sex, HTN, DM, and perforation size had no significant impact on the incidence of surgical failure. Regarding age, although the failure rate was high in the elderly, it was not statistically significant. Moreover, all fat myringoplasty procedures performed on a super-aged population > 80 years of age were successful.

Looking at the re-perforation cases in our study, four of them (44.4%) exhibited a gap at the fat graft site over time due to the transplanted fat being insufficient. Three of the nine cases with re-perforation (33.3%) had postoperative MRSA infection, while the others (22.2%) exhibited extrusion of transplanted fat after nasal blowing or coughing. In conclusion, the factors affecting re-perforation in our study were insufficient fat graft, postoperative MRSA infection, and nasal blowing.

### Hearing according to age and hearing change after fat myringoplasty

**Fig. 2:**
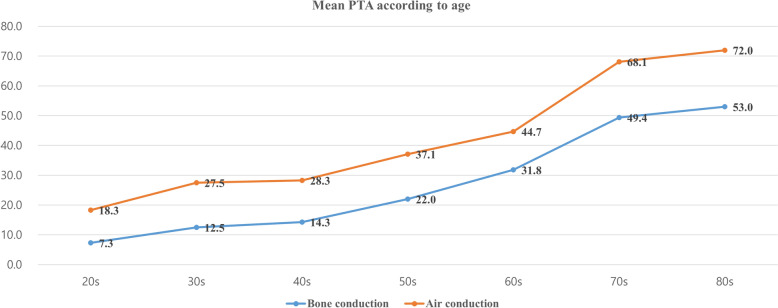
Hearing according to age in patients with chronic otitis media. The upper line represents the pure tone audiometry air conduction threshold of patients with chronic otitis media, and the lower line is the bone conduction threshold. The difference between the two lines is an air–bone gap, which occurs when patients with chronic otitis media experience conductive hearing loss due to perforation of the eardrum. People older than 60 years have a hearing threshold of > 40 dB, which requires hearing aids

We next investigated the hearing changes according to age in patients with COM. Air–bone gaps were observed because perforation of the eardrum interfered with sound transmission, while closing the perforation with a fat graft improves hearing. Furthermore, patients with COM aged older than 60 years exhibited hearing loss of > 40 dB, which may indicate the need for a hearing aid (Fig. 2).

We also analyzed hearing changes after fat myringoplasty. Postoperative PTA was performed in 67 of 141 patients, including 24 elderly and 43 adult patients. We investigated BC, AC, and ABG before and after fat myringoplasty according to age group.

**Fig. 3:**
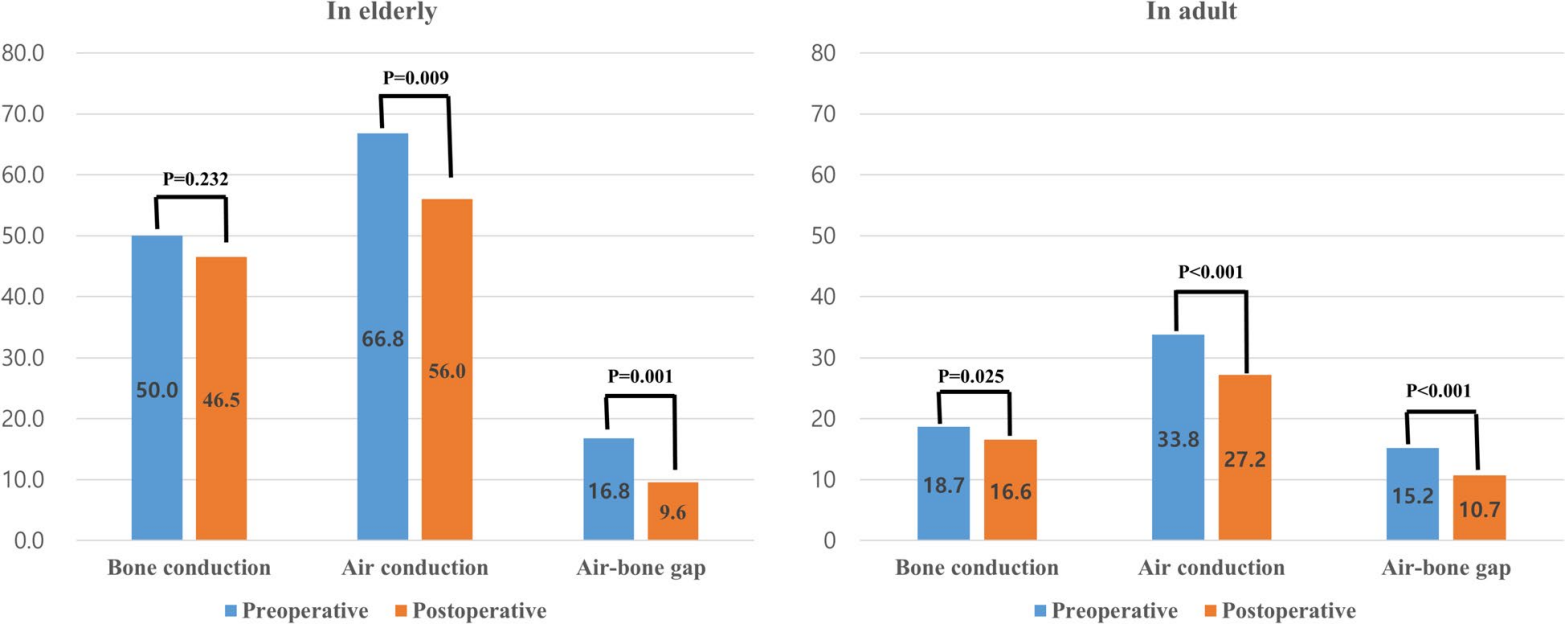
Change in pure tone audiometry before and after fat myringoplasty according to age group. In both the elderly and adult groups, the hearing improved after surgery as the air–bone gap and air conduction threshold decreased

In the elderly group, the mean BC, AC, and ABG on preoperative PTA were 50.0 ± 15.8, 66.8 ± 18.5, and 16.8 ± 6.6 dB, respectively. In the adult group, the mean BC, AC, and ABG on preoperative PTA were 18.7 ± 15.2, 33.8 ± 19.1, and 15.2 ± 8.2 dB, respectively. Both the elderly and adult patients showed significant improvements in hearing after surgery (Fig. 3). In other words, fat myringoplasty in the elderly not only reduces inflammation that occurs when wearing hearing aids but can also be expected to improve hearing.

## Discussion

Hearing loss is the most common sensory deficit in the elderly and is becoming a severe social and health problem. In particular, in the elderly, hearing loss can impair the exchange of information, thus significantly impacting everyday life, causing loneliness, isolation, dependence, frustration, and communication disorders [10].

Hearing loss in older people is a natural part of the aging process. As humans age, degeneration within the inner ear and along the nerve pathways to the brain can result in hearing loss [11]. Because hair cells do not regenerate or regrow, this damage is permanent [12]. In our study, people younger than 60 years of age had relatively normal hearing (mean BC ≤ 25 dB), while those older than 60 years exhibited hearing loss (mean BC > 25). In particular, elderly people older than 70 years showed moderate hearing loss (40 < mean BC ≤ 55 dB) necessitating a hearing aid for hearing rehabilitation.

In older people with COM, surgery is important for three reasons. First, correction of conductive hearing loss caused by perforation of the tympanic membrane may obviate the need for a hearing aid or at least increase its effectiveness [13]. Indeed, our results demonstrated that hearing improvement decreased the air–bone gap following surgery. Second, correction of tympanic membrane perforation may prevent acceleration of cochlear damage in older people. Damage to the cochlea may result from continuous exposure of the cochlea to external factors such as water, sweat, or discharge of the external ear canal through tympanic membrane perforation [14]. Third, correction of tympanic membrane perforation may reduce bacterial or fungal infection in the middle or external ear aggravated by the use of a hearing aid.

Surgery was previously considered to be inadvisable in older patients with COM because of the high incidence of graft failure and the greater risk of pulmonary, cardiovascular, and other complications [15, 16]; in this situation, fat myringoplasty, as an easy, simple, and minimally invasive procedure without complications, represents a good alternative. Additionally, fat myringoplasty can be performed under local anesthesia, has a short operation time, favorable hearing results, and a good success rate [17].

In our study, the average operation time was 20 min, and most of the patients were discharged on the same day, with no complications such as dizziness or iatrogenic hearing loss after fat myringoplasty. Regarding the success rate of surgery, although the older patients had lower success rates than the younger patients, there was no statistical difference between the two groups. Moreover, fat myringoplasty was successful in all seven patients who were older than 80 years. This is considered strong evidence that age does not correlate with the outcome of fat myringoplasty. We also found that hypertension, diabetes, and the size of the perforation did not affect the surgical outcome.

Among the 141 included patients, nine experienced surgery failure for the following reasons: three due to postoperative MRSA infection, two due to fat extrusion by nasal blowing or coughing, and four due to perforation dehiscence due to insufficient fat tissue. In other words, our findings suggest that external factors, such as infection, nasal blowing, cough, and insufficient grafted fat tissue, affect surgical outcomes more than intrinsic factors such as age or underlying diseases.

In our study, older people demonstrated hearing improvement after fat myringoplasty, as evidenced by improvements in BC, AC, and ABG. Fat myringoplasty, which is non-invasive and does not require middle ear exploration, ossicle manipulation, or bone drilling, was thought to contribute to the prevention of an increase in the BC threshold (indicative of cochlear damage). In older people, fat myringoplasty also improved AC and speech discrimination by reducing the ABG caused by tympanic membrane perforation. This is expected to improve their quality of life by increasing the effectiveness of the hearing aid.

Fat myringoplasty has limited surgical indication in patients with COM. In particular, if the perforation size is large, the success rate of surgery decreases [18]. Because, in cases with a large perforation, the fat graft may escape into the middle ear or into the external auditory canal more easily and may not undergo proper epithelialization [19]. Furthermore, the inherent property of fat thickness could impair the vibration of the tympanic membrane, and may induce less ABG closure than tympanoplasty with fascia or perichondrium [20].

Nevertheless, fat myringoplasty has a definite advantage compared to classical tympanoplasty in patients with COM, including the use of local anesthesia, short operation time, easy technique, and fewer complications [21]. Therefore, fat myringoplasty may be the most effective method for elderly people who require surgery for COM to improve hearing, control middle ear inflammation, and wear hearing aids.

### Limitation

This study has several limitations that warrant discussion. First, the difference in the occurrence of re-perforation between the two groups according to age was not significant (p = 0.072), although this may change with a greater number of samples. Second, as the incidence of HTN varied between the elderly and control adult groups, they cannot be said to be completely the same group except for age. Finally, to strengthen the validity of these findings, future studies would benefit from a larger sample size and a more narrowly defined age range.

## Conclusion

Surgical strategies for elderly patients should be minimally invasive, safe, and easy, with a short operation time and fewer complications. Therefore, fat myringoplasty may represent a good option for elderly patients with COM. Although age and underlying conditions play significant roles in the healing process, our results suggest that external factors such as infection, nasal blowing, cough, and insufficient grafted fat tissue have a similarly significant impact on surgical outcomes in elderly patients with COM as they do in adults. In conclusion, the decision to perform surgery in elderly patients with COM should be based on a comprehensive assessment of the patient’s overall health status, hearing, use of hearing aids, and the indications for surgery.

## Data Availability

No datasets were generated or analysed during the current study.

## Abbreviations

COM: Chronic otitis media
PTA: Pure tone audiometry
BC: Bone conduction
AC: Air conduction
ABG: Air–bone gap
MRSA: Methicillin-resistant *Staphylococcus aureus*
